# Implications for quantifying early life growth trajectories of term‐born infants using INTERGROWTH‐21st newborn size standards at birth in conjunction with World Health Organization child growth standards in the postnatal period

**DOI:** 10.1111/ppe.12880

**Published:** 2022-05-16

**Authors:** Nandita Perumal, Eric O. Ohuma, Andrew M. Prentice, Prakesh S. Shah, Abdullah Al Mahmud, Sophie E. Moore, Daniel E. Roth

**Affiliations:** ^1^ Department of Global Health and Population Harvard TH Chan School of Public Health Boston Massachusetts USA; ^2^ Centre for Global Child Health Peter Gilgan Centre for Research and Learning The Hospital for Sick Children Toronto Ontario Canada; ^3^ Maternal, Adolescent, Reproductive and Child Health Centre, Department of Infectious Disease Epidemiology London School of Hygiene and Tropical Medicine London UK; ^4^ MRC Unit The Gambia at the London School of Hygiene and Tropical Medicine Fajara The Gambia; ^5^ Department of Pediatrics Mount Sinai Hospital & the University of Toronto Toronto Ontario Canada; ^6^ International Centre for Diarrheal Disease Research, Bangladesh (icddr,b) Dhaka Bangladesh; ^7^ Department of Women and Children’s Health King’s College London London UK; ^8^ Department of Pediatrics Hospital for Sick Children & the University of Toronto Toronto Ontario Canada; ^9^ Department of Nutritional Sciences University of Toronto Toronto Ontario Canada

**Keywords:** anthropometry, INTERGROWTH‐21st newborn size standards, growth, newborn, term‐born, World Health Organization child growth standards

## Abstract

**Background:**

The INTERGROWTH‐21st sex and gestational age (GA) specific newborn size standards (IG‐NS) are intended to complement the World Health Organization Child Growth Standards (WHO‐GS), which are not GA‐specific. We examined the implications of using IG‐NS at birth and WHO‐GS at postnatal ages in longitudinal epidemiologic studies.

**Objectives:**

The aim of this study was to quantify the extent to which standardised measures of newborn size and growth are affected when using WHO‐GS versus IG‐NS at birth among term‐born infants.

**Methods:**

Data from two prenatal trials in Bangladesh (*n* = 755) and The Gambia (*n* = 522) were used to estimate and compare size at birth and growth from birth to 3 months when using WHO‐GS only (‘WHO‐GS’) versus IG‐NS at birth and WHO‐GS postnatally (‘IG‐NS’). Mean length‐for‐age (LAZ), weight‐for‐age (WAZ) and head circumference‐for‐age (HCAZ), and the prevalence of undernutrition (stunting: LAZ < −2SD; underweight: WAZ < −2SD; and microcephaly: HCAZ < −2SD) were estimated overall and by GA strata [early‐term (37^0/7^–38^6/7^), full‐term (39^0/7^–40^6/7^) and late‐term (41^0/7^–43^0/7^)]. We used Bland–Altman plots to compare continuous indices and Kappa statistic to compare categorical indicators.

**Results:**

At birth, mean LAZ, WAZ and HCAZ, and the prevalence of undernutrition were most similar among newborns between 39 and 40 weeks of GA when using WHO‐GS versus IG‐NS. However, anthropometric indices were systematically lower among early‐term infants and higher among late‐term infants when using WHO‐GS versus IG‐NS. Early‐term and late‐term infants demonstrated relatively faster and slower growth, respectively, when using WHO‐GS versus IG‐NS, with the direction and magnitude of differences varying between anthropometric indices. Individual‐level differences in attained size and growth, when using WHO‐GS versus IG‐NS, were greater than 0.2 SD in magnitude for >60% of infants across all anthropometric indices.

**Conclusions:**

Using IG‐NS at birth with WHO‐GS postnatally is acceptable for full‐term infants but may give a misleading interpretation of growth trajectories among early‐ and late‐term infants.

**Synopsis**

1 **Study question**

What are the implications of using gestational age (GA) and sex‐specific INTERGROWTH‐21st newborn size standards (IG‐NS) at birth and World Health Organization Child Growth Standards (WHO‐GS) at postnatal ages for growth trajectories in term‐born infants?

2 **What is already known?**

The IG‐NS were constructed using methods that conceptually and methodologically complement the WHO‐GS postnatally, such that the medians of standardised anthropometric indices at birth align at 40 weeks of GA. Therefore, the IG‐NS have been promoted for use in conjunction with WHO‐GS in clinical and research settings.

3 **What this study adds?**

Using IG‐NS at birth and WHO‐GS postnatally is acceptable for full‐term infants (39^0/7^–40^6/7^ weeks) but gives a misleading interpretation of size and growth trajectories of early‐term (37^0/7^–38^6/7^ weeks) and late‐term (41^0/7^–43^0/7^ weeks) infants.

## 1 BACKGROUND

The World Health Organization Child Growth Standards (WHO‐GS) are the most widely used normative tool to evaluate postnatal child growth.^1^ These standards are based on a multi‐ethnic cohort of term‐born children (37–42 weeks of gestational age (GA)) and are commonly applied in epidemiological research to estimate age‐ and sex‐standardised anthropometric indices of nutritional status.^2^ Although the WHO‐GS are used to assess child nutritional status from birth up until 5 years of age, these standards do not account for variations in newborn size due to heterogeneity in GA at birth.^2^ The International Fetal and Newborn Growth Consortium for the 21st Century (INTERGROWTH‐21st) newborn size standards (IG‐NS) for infants born between 33 and 42 weeks of GA provide a complementary tool to derive standardised indices of newborn size that takes into account the GA at birth.^3^ The IG‐NS were constructed using participant inclusion criteria and study methods to conceptually and methodologically complement the WHO‐GS postnatally. It is therefore reasonable for researchers and clinicians to use both standards in a manner that most accurately reflects the nutritional status of a child relative to the standard population at a given chronological age and while appropriately considering GA at birth and sex.

The IG‐NS have been promoted to replace previous newborn size references and complement the WHO‐GS in clinical settings and in epidemiologic research.^4^, ^5^ However, in the context of longitudinal epidemiologic studies or repeated cross‐sectional surveys of postnatal child growth, there are unclear implications of using IG‐NS at birth in conjunction with the WHO‐GS at subsequent postnatal ages. The combined use of IG‐NS at birth and WHO‐GS postnatally assumes that the two standards are interchangeable where they overlap. Therefore, among term‐born children (i.e. 37–42 weeks of GA at birth), *z*‐scores for birth size derived using the two standards should be very similar. The IG‐NS investigators demonstrated that the medians of the IG‐NS and WHO‐GS standards align for infants at 40 weeks of GA^3^; however, there has been no evidence to demonstrate that *z*‐scores across the distribution of newborn weights or lengths similarly align between the two standards. If the standards are not well aligned across their entire distributions, then the combined use of IG‐NS (to standardise weights, lengths or head circumferences at birth) with WHO‐GS (to standardise postnatal child lengths, weights or head circumferences) may result in artefactual, rather than biological, within‐child changes in *z*‐scores between birth and a postnatal time point in clinical assessments and in longitudinal studies of child growth.

The aim of this study was to quantify the extent to which standardised measures of newborn size and growth in early infancy are affected when using different standards at birth in two birth cohorts from Bangladesh and The Gambia. First, we compared the use of WHO‐GS versus IG‐NS to estimate mean anthropometric indices at birth, including length‐for‐age (LAZ), weight‐for‐age (WAZ) and head circumference‐for‐age *z*‐scores (HCAZ), and the prevalence of anthropometric indicators, including stunting (LAZ < −2 SD), underweight (WAZ < −2SD) and microcephaly (HCAZ < −2SD). Second, we estimated the changes in LAZ, WAZ and HCAZ from birth to 3 months of age when using IG‐NS at birth in conjunction with WHO‐GS postnatally compared with using WHO‐GS both at birth and postnatally.

## 2 METHODS

### 2.1 Study population

This is a secondary analysis of data from term‐born infants in the Maternal Vitamin D for Infant Growth (MDIG) trial (Clinical Trial number: NCT01924013) and the Early Nutrition and Immune Development (ENID) trial (Trial Registration: ISRCTN49285450).^6^, ^7^ The MDIG and ENID trials were both previously described in detail.^6^, ^7^, ^8^, ^9^ Briefly, the MDIG trial was a randomised, placebo‐controlled, double‐blinded, dose‐ranging trial in Dhaka, Bangladesh, in which women (*n* = 1298) were randomly assigned at mid‐gestation (17–24 weeks of GA) to one of the five groups of vitamin D supplementation. The ENID trial was a 2 × 2 × 2 factorial design randomised controlled trial conducted in the West Kiang region of the Gambia in which women (*n* = 875) who were between 10 and 20 weeks of pregnancy were randomly assigned to one of four prenatal nutrition interventions arms. Mothers and infants were followed at multiple time points throughout pregnancy and in the postpartum period in both trials. We used data from these two studies to assess the consistency of inferences across populations with differing nutritional status.

Because variation in GA at birth is most likely to influence growth trajectories in early life and has diminishing impact over time,^10^, ^11^ only anthropometric data collected at birth (within 48 h) and at the 3‐month postnatal age visit (median days [interquartile range]: MDIG 91 days [91,92]; ENID: 95 days [92.5, 97]) for infants born at term (i.e. born between 259 to 300 days GA) were included in this study. Data from all eligible infants, irrespective of maternal group randomisation, were included due to a lack of treatment effect in MDIG and ENID trials on infant anthropometric outcomes.^8^, ^12^ Data from infants born outside the specified term GA range, stillbirths and infants with any congenital anomalies were excluded.

### 2.2 Gestational age assessment

In both MDIG and ENID studies, ultrasound‐based assessments were used to measure GA.^6^, ^7^ In MDIG, ultrasounds in the second trimester were used to confirm or revise GA by date of last menstrual period according to a standard algorithm.^8^ In ENID, only ultrasound‐based GA assessments were used.

### 2.3 Anthropometric measures and indices

Anthropometry was assessed using standardised procedures by trained personnel at birth and 3 months.^7^, ^8^, ^9^ Infant weight was measured using a digital scale to the nearest 10 g (with additional precision to nearest 5 g among infants <10 kg in the MDIG trial). Crown‐to‐heel length and head circumference were measured with 1‐mm precision. Measurements were obtained independently by two study personnel and showed high interrater reliability in the MDIG trial. Measurements in the ENID trial were taken in triplicate, by a single observer. Means of the final pair or triplicate values were used in analyses. Length, weight and head circumference at birth were expressed as GA‐ and sex‐standardised *z*‐scores according to IG‐NS or postnatal age‐ and sex‐standardised *z*‐scores using the WHO‐GS. At 3‐month postnatal age, only the WHO‐GS were used to derive age‐ and sex‐standardised *z*‐scores for anthropometric measures.

### 2.4 Statistical analysis

In cross‐sectional analyses of birth size measurements, we estimated and compared mean LAZ, WAZ or HCAZ and the proportion of newborns classified as being undernourished (i.e. stunted, underweight or microcephalic) using WHO‐GS versus IG‐NS (whereby the latter was considered the reference for comparison as it accounts for variations in GA at birth). We used Bland–Altman plots to assess the distribution of individual‐level differences in LAZ, WAZ or HCAZ and the Kappa statistic to estimate the degree of within‐infant concordance in stunting, underweight or microcephaly classification when using WHO‐GS versus IG‐NS at birth. We also estimated sensitivity and specificity of indicators of undernutrition at birth using WHO‐GS versus IG‐NS.

Using WHO‐GS at birth and postnatally (‘WHO‐GS’) or IG‐NS at birth combined with WHO‐GS postnatally (‘IG‐NS’), we estimated infant growth in the first 3‐month postnatal age by quantifying: (i) mean change in LAZ, WAZ or HCAZ from birth to 3 months, referred as to ΔLAZ, ΔWAZ and ΔHCAZ, respectively; and (ii) mean LAZ, WAZ or HCAZ at 3 months conditional on size at birth, respectively. The latter, ‘conditional measures’ of growth, were the infant‐level model residuals from the linear regression of size at 3 months on size at birth. A positive residual indicates greater than expected growth given size at birth, whereas a negative residual indicates lower than expected growth given size at birth, relative to other infants in the cohort. For any index based on conditional growth, the overall group mean is always zero; therefore, mean conditional measures are only reported by GA strata. We compared WHO‐GS and IG‐NS with respect to mean changes in *z*‐scores from birth to 3 months and mean conditional measures of size at 3 months using Bland–Altman plots to visualise the distributions of individual‐level differences between WHO‐GS and IG‐NS.

In further analyses, we classified infants according to (a) individual‐level differences in *z*‐scores at birth derived using WHO‐GS versus IG‐NS; or (b) differences in ΔLAZ, ΔWAZ and ΔHCAZ using WHO‐GS versus IG‐NS at birth. Classifications were based on whether the absolute value of the difference exceeded a priori thresholds of 0.20 SD, 0.32 SD and 0.50 SD. An effect size of 0.2 SD is a commonly observed magnitude of change in anthropometric indices (namely LAZ) due to nutrition‐specific interventions.^13^ The threshold value of 0.32 SD is the *z*‐score difference between the 5th (1.64 SD) and 2.5th (1.96 SD) percentile of a normal distribution, which was the stricter criterion used to assess comparability of data across sites by the INTERGROWTH‐21st consortium.^14^ Finally, we selected 0.50 SD as a threshold value as these are the criteria used by the INTERGROWTH‐21st consortium and the WHO multicentre growth reference study group for the WHO child growth standards as the allowable average difference in population mean *z*‐scores to assess acceptability of pooling data from different sites.

All analyses were stratified by GA [i.e. early‐term (37^0/7^ to 38^6/7^ weeks), full‐term (39^0/7^ to 40^0/7^ weeks) and late‐term (41^0/7^ to 43^0/7^ weeks)] within the broad category of ‘term’ gestation to assess the extent to which overall heterogeneity in early postnatal growth trajectories may be due to variation in GA at birth. Analysis for each cohort was conducted independently using Stata version 14.0 and R package.

### 2.5 Missing data

Only infants with GA and at least one anthropometric measure were eligible for inclusion. Given the methodological focus of this study, we did not use multiple imputation methods to impute any anthropometric values and there were no missing values for age or sex, which were the only covariates considered in the analysis. The number of infants was consistent within each anthropometric measure (i.e. length, weight or head circumference) for primary comparisons of anthropometric indices and indicators using WHO‐GS versus IG‐NS.

### 2.6 Ethics approval

Ethics approval for the use of de‐identified secondary data from the two trials was obtained from the Hospital for Sick Children Research Ethics Board, Toronto, Canada (Protocol # 1000057464).

## 3 RESULTS

Gestational age and at least one anthropometric measure at birth were available for 837 infants from the MDIG study and 681 infants from the ENID study. After exclusion of infants with congenital anomalies (*n* = 28 in MDIG), preterm‐born infants (*n* = 53 in MDIG and *n* = 9 in ENID) and infants for whom birth anthropometry was taken after 48 h (*n* = 150 in ENID), data from 755 infants from the MDIG study and 522 infants from the ENID study were included in the final analytical sample. Baseline maternal characteristics of infants included in this study differed between the cohorts (Table S1).

### 3.1 Cross‐sectional comparisons using WHO‐GS versus IG‐NS at birth

On average, newborns were shorter and lighter in MDIG compared with ENID, but HCAZs were similar between the two cohorts (Table 1). Mean *z*‐scores differed between WHO‐GS and IG‐NS for all indices and across all GA strata in both cohorts, except for mean LAZ overall in ENID (Table 1; Figure S1). Mean LAZ, WAZ and HCAZ were all substantially lower among early‐term infants and higher among late‐term infants using WHO‐GS versus IG‐NS in both cohorts (Table 1). The overall mean differences between WHO‐GS and IG‐NS in LAZ, WAZ and HCAZ were generally small in both cohorts (Figure S2).

**TABLE 1:** Birth length‐for‐age, weight‐for‐age and head circumference‐for‐age *z*‐scores among term‐born infants using the INTERGROWTH‐21st Neonatal Standards compared with the WHO Child Growth Standards.

Anthropometric indices	Birth						
	*n*	World Health Organization Growth Standards (WHO‐GS)		INTERGROWTH‐21st neonatal standards (IG‐NS)		Mean differences in *z*‐scores using WHO‐GS compared with IG‐NS	
		Mean (SD)	Range: min, max	Mean (SD)	Range: min, max	Mean (SD)	Range: min, max
MDIG cohort							
Length‐for‐age *z*‐score							
Overall, term births (37^0/7^ to 43^0/7^)	744	−1.05 (0.99)	−5.67, 1.94	−0.92 (1.00)	−4.57, 2.24	−0.12 (0.43)	−1.68, 1.13
Early term (37^0/7^ to 38^6/7^)	286	−1.37 (0.98)	−5.58, 0.96	−0.83 (1.00)	−4.14, 1.57	−0.54 (0.25)	−1.68, −0.14
Full term (39^0/7^ to 40^6/7^)	396	−0.87 (0.97)	−5.67, 1.94	−0.93 (1.02)	−4.57, 2.24	0.06 (0.23)	−1.23, 0.52
Late term (41^0/7^ to 43^0/7^)	62	−0.64 (0.77)	−2.44, 1.30	−1.26 (0.88)	−3.37, 1.03	0.62 (0.19)	0.27, 1.13
Weight‐for‐age *z*‐score							
Overall, term births (37^0/7^ to 43^0/7^)	753	−1.25 (0.79)	−3.96, 0.90	−1.22 (0.85)	−3.26, 1.68	−0.04 (0.40)	−1.01, 1.07
Early term (37^0/7^ to 38^6/7^)	292	−1.41 (0.81)	−3.96, 0.85	−0.97 (0.84)	−3.16, 1.68	−0.44 (0.23)	−1.01, −0.09
Full term (39^0/7^ to 40^6/7^)	398	−1.17 (0.76)	−3.20, 0.90	−1.33 (0.81)	−3.23, 1.18	0.16 (0.19)	−0.34, 0.57
Late term (41^0/7^ to 43^0/7^)	63	−1.05 (0.75)	−2.98, 0.53	−1.68 (0.79)	−3.26, 0.16	0.63 (0.17)	0.28, 1.07
Head circumference‐for‐age *z*‐score							
Overall, term births (37^0/7^ to 43^0/7^)	748	−0.90 (0.89)	−3.78, 1.76	−0.62 (0.95)	−3.24, 2.03	−0.28 (0.40)	−1.15, 1.01
Early term (37^0/7^ to 38^6/7^)	290	−1.08 (0.89)	−3.78, 1.12	−0.41 (0.93)	−3.16, 2.03	−0.67 (0.20)	−1.15, −0.34
Full term (39^0/7^ to 40^6/7^)	395	−0.83 (0.87)	−3.51, 1.76	−0.72 (0.92)	−3.18, 1.91	−0.10 (0.19)	−0.51, 0.36
Late term (41^0/7^ to 43^0/7^)	63	−0.58 (0.86)	−2.88, 1.21	−1.01 (1.00)	−3.24, 1.14	0.43 (0.21)	0.07, 1.01
ENID cohort							
Length‐for‐age *z*‐score							
Overall, term births (37^0/7^ to 43^0/7^)	518	−0.10 (1.02)	−4.42, 4.87	−0.11 (1.12)	−3.80, 4.27	0.03 (0.43)	−1.14, 1.03
Early term (37^0/7^ to 38^6/7^)	79	−0.56 (0.98)	−4.43, 1.80	0.05 (1.04)	−3.38, 2.62	−0.61 (0.23)	−1.14, 0.15
Full term (39^0/7^ to 40^6/7^)	289	−0.11 (0.99)	−4.19, 4.29	−0.06 (1.08)	−3.80, 3.80	−0.04 (0.24)	−0.67, 0.49
Late term (41^0/7^ to 43^0/7^)	150	0.16 (1.02)	−2.41, 4.87	−0.30 (1.22)	−3.31, 4.27	0.49 (0.23)	−0.03, 1.03
Weight‐for‐age *z*‐score							
Overall, term births (37^0/7^ to 43^0/7^)	519	−0.67 (0.90)	−3.23, 1.76	−0.86 (0.96)	−3.13, 1.95	0.18 (0.37)	−1.06, 0.98
Early term (37^0/7^ to 38^6/7^)	78	−1.07 (0.93)	−3.23, 0.93	−0.66 (1.00)	−2.71, 1.53	−0.41 (0.20)	−1.06, 0.09
Full term (39^0/7^ to 40^6/7^)	290	−0.73 (0.84)	−2.77, 1.76	−0.87 (0.91)	−2.71, 1.95	0.14 (0.20)	−0.47, 0.57
Late term (41^0/7^ to 43^0/7^)	151	−0.37 (0.90)	−2.63, 1.53	−0.94 (1.03)	−3.13, 1.29	0.56 (0.18)	0.14, 0.98
Head circumference‐for‐age *z*‐score							
Overall, term births (37^0/7^ to 43^0/7^)	519	−0.88 (1.09)	−3.70, 2.16	−0.84 (1.13)	−3.82, 2.60	−0.04 (0.40)	−1.15, 0.89
Early term (37^0/7^ to 38^6/7^)	79	−1.36 (1.01)	−3.04, 0.96	−0.71 (1.03)	−2.56, 1.58	−0.65 (0.18)	−1.15, 0.35
Full term (39^0/7^ to 40^6/7^)	289	−0.91 (1.06)	−3.70, 2.14	−0.80 (1.11)	−3.36, 2.60	−0.11 (0.21)	−0.67, 0.33
Late term (41^0/7^ to 43^0/7^)	151	−0.56 (1.09)	−3.37, 2.16	−0.97 (1.21)	−3.82, 2.10	0.41 (0.21)	−0.04, 0.89

**FIGURE 1:**
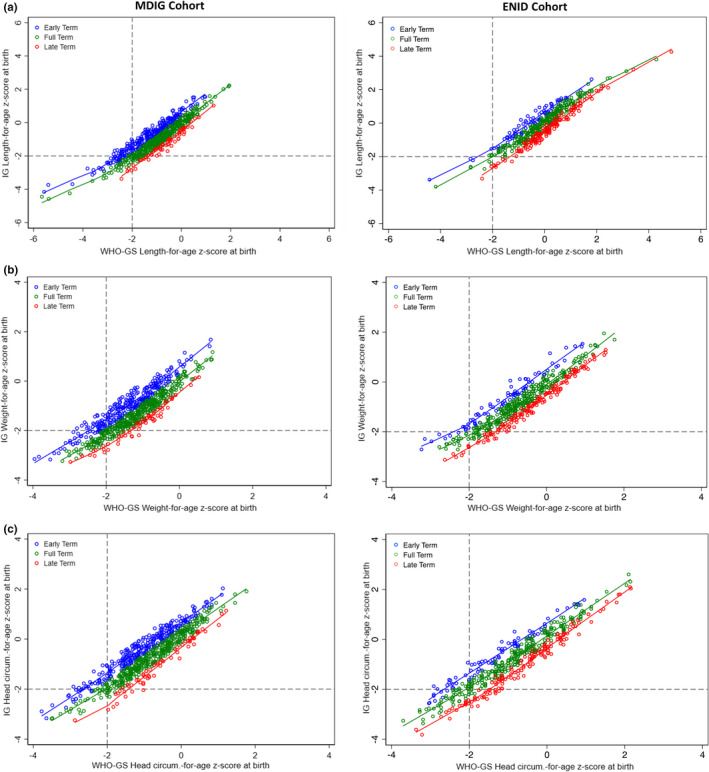
Birth length‐for‐age ‐(A), weight‐for‐age (B) and head circumference‐for‐age (C) *z*‐scores among term‐born children using the World Health Organization Child Growth Standards (WHO‐GS) compared with the INTERGROWTH‐21st newborn size standards (IG‐NS). The dashed lines indicate the cut‐off values for anthropometric indicators

Comparing WHO‐GS versus IG‐NS, the proportion of children classified as being stunted at birth was similar overall in both cohorts (MDIG: 15% vs. 13%; ENID: 3.1% vs. 3.8%), the prevalence of underweight was similar in MDIG (18% vs. 17%) but lower in ENID (7.5% vs. 12%), and the prevalence of microcephaly was higher in MDIG (10% vs. 7.6%) but the same in ENID (Table 2). Using WHO‐GS (vs. IG‐NS), the proportion of newborns classified as being undernourished at birth in both cohorts was always higher among early‐term infants, but lower among late‐term infants (Table 2; Figure 1). Using IG‐NS as the reference, the overall specificity of classifying undernourished infants using WHO‐GS was high (>90%) across all anthropometric indicators in both cohorts. However, by GA strata, WHO‐GS (vs. IG‐NS) had high sensitivity but lower specificity in identifying undernutrition among early‐term infants, and low sensitivity and high specificity among late‐term infants (Table 2). The discordance in classification of stunting, underweight and microcephaly at the individual level was marked by the generally lower Kappa statistic among early‐ and late‐term infants relative to the concordance overall or within the full‐term GA strata (Table 2).

**TABLE 2:** Prevalence of stunting (length‐for‐age *z*‐score < −2), underweight (weight‐for‐age *z*‐score < −2) and microcephaly (head circumference‐for‐age *z*‐score < −2) at birth among term‐born children using the WHO‐GS versus INTERGROWTH‐21st Neonatal Standards

Anthropometric indices	Prevalence of undernutrition (<−2SD)			Sensitivity^a^ % (95% CI)	Specificity^a^ % (95% CI)	Kappa Statistic (95% CI)
	*n*	WHO‐GS *n* (%)	IG‐NS *n* (%)			
MDIG Cohort						
Length‐for‐age *z*‐score <−2 SD						
Overall (37^0/7^ to 43^0/7^)	744	113 (15)	99 (13)	76 (66, 84)	94 (92, 96)	0.66 (0.58, 0.74)
Early term (37^0/7^ to 38^6/7^)	286	72 (25)	36 (13)	100 (90, 100)	86 (80.6, 90)	0.60 (0.49, 0.71)
Full term (39^0/7^ to 40^6/7^)	396	37 (9.3)	52 (13)	67 (53, 80)	99 (98, 100)	0.76 (0.66, 0.86)
Late term (41^0/7^ to 43^0/7^)	62	4 (6.5)	11 (18)	36 (11, 69)	100 (93, 100)	0.48 (0.18, 0.79)
Weight‐for‐age *z*‐score <−2 SD						
Overall (37^0/7^ to 43^0/7^)	753	136 (18)	131 (17)	75 (67, 82)	94 (92, 96)	0.68 (0.61, 0.75)
Early term (37^0/7^ to 38^6/7^)	292	67 (23)	29 (9.9)	100 (88, 100)	86 (81, 90)	0.54 (0.42, 0.66)
Full term (39^0/7^ to 40^6/7^)	398	60 (15)	81 (20)	74 (63, 83)	100 (99, 100)	0.82 (0.75, 0.89)
Late term (41^0/7^ to 43^0/7^)	63	9 (14)	21 (33)	43 (22, 66.)	100 (92, 100)	0.50 (0.28, 0.72)
Head circumference‐for‐age *z*‐score <−2 SD						
Overall (37^0/7^ to 43^0/7^)	748	78 (10)	57 (7.6)	75 (62, 86)	95 (93, 96)	0.60 (0.50, 0.70)
Early term (37^0/7^ to 38^6/7^)	290	43 (15)	17 (5.6)	100 (81, 100)	91 (86, 94)	0.53 (0.37, 0.68)
Full term (39^0/7^ to 40^6/7^)	395	34 (8.6)	29 (7.3)	86 (68, 96)	98 (95, 99)	0.76 (0.66, 0.89)
Late term (41^0/7^ to 43^0/7^)	63	1 (1.6)	11 (18)	9.1 (0.23, 41)	100 (93, 100)	0.14 (−0.11, 0.39)
ENID cohort						
Length‐for‐age *z*‐score <−2 SD						
Overall (37^0/7^ to 43^0/7^)	518	16 (3.1)	20 (3.8)	55 (32, 77)	99 (98, 100)	0.60 (0.41, 0.79)
Early term (37^0/7^ to 38^6/7^)	79	4 (5.1)	3 (3.8)	100 (29, 100)	98 (93, 100)	0.85 (0.56, 1.00)
Full term (39^0/7^ to 40^6/7^)	289	10 (3.5)	8 (2.8)	75 (35, 97)	99 (96, 100)	0.65 (0.40, 0.91)
Late term (41^0/7^ to 43^0/7^)	150	2 (1.3)	9 (5.9)	22 (2.8, 60)	100 (96, 100)	0.35 (−0.01, 0.71)
Weight‐for‐age *z*‐score <−2 SD						
Overall (37^0/7^ to 43^0/7^)	519	39 (7.5)	62 (12)	53 (40, 66)	99 (97, 100)	0.62 (0.50, 0.73)
Early term (37^0/7^ to 38^6/7^)	78	13 (17)	7 (8.9)	100 (59, 100)	92 (83, 97)	0.65 (0.42, 0.91)
Full term (39^0/7^ to 40^6/7^)	290	18 (6.2)	30 (10)	60 (41, 77)	100 (99, 100)	0.73 (0.58, 0.87)
Late term (41^0/7^ to 43^0/7^)	151	8 (5.3)	25 (17)	32 (15, 53)	100 (97, 100)	0.44 (0.23, 0.65)
Head circumference‐for‐age *z*‐score <−2 SD						
Overall (37^0/7^ to 43^0/7^)	519	87 (17)	88 (17)	73 (62, 82)	95 (92, 97)	0.68 (0.59, 0.76)
Early term (37^0/7^ to 38^6/7^)	79	22 (28)	8 (10)	100 (63, 100)	80 (69, 89)	0.45 (0.23, 0.67)
Full term (39^0/7^ to 40^6/7^)	289	49 (17)	43 (15)	93 (81, 99)	96 (93, 98)	0.85 (0.76, 0.93)
Late term (41^0/7^ to 43^0/7^)	151	16 (11)	37 (24)	43 (27, 61)	100 (97, 100)	0.54 (0.37, 0.70)

^a^ Sensitivity and specificity of WHO‐GS relative to IG‐NS, considered the reference method in this analysis.

### 3.2 Comparisons of growth metrics using WHO‐GS versus IG‐NS at birth

At 3 months, mean LAZ, WAZ and HCAZ were derived using WHO‐GS only (Table S1). Mean ΔLAZ, ΔWAZ and ΔHCAZ differed significantly when derived using WHO‐GS at birth and 3 months compared with using IG‐NS at birth with WHO‐GS at 3 months (‘IG‐NS’); however, the direction and magnitude of those differences varied substantially by cohort, anthropometric measure and GA stratum (Table 3). In both cohorts, differences between WHO‐GS and IG‐NS were amplified in the early‐ and late‐term infant strata, and the direction of change was often discrepant (Table 3). Bland–Altman plots of changes in size from birth to 3 months showed that the systematic differences between WHO‐GS and IG‐NS were greatest for ΔLAZ and ΔHCAZ in MDIG and for ΔWAZ in ENID (Figure S3).

**TABLE 3:** Mean change (Δ) in length‐for‐age, weight‐for‐age and head circumference‐for‐age *z*‐scores among term‐born children using the WHO‐GS or INTERGROWTH‐21st Neonatal Standards at birth in conjunction with WHO‐GS at 3 months of age

Anthropometric indices	Mean change in *z*‐scores (Δ) from birth to 3 months of age				
	*n*	WHO‐GS at birth and WHO‐GS at 3 months of age		IG‐NS at birth and WHO‐GS at 3 months of age	
		Mean Δ (SD)	Range: min, max	Mean Δ (SD)	Range: min, max
MDIG Cohort					
Length‐for‐age *z*‐score					
Overall, term births (37^0/7^ to 43^0/7^)	709	0.22 (0.68)	−2.73, 4.38	0.09 (0.75)	−3.50, 2.97
Early term (37^0/7^ to 38^6/7^)	273	0.37 (0.72)	−2.73, 4.38	−0.17 (0.75)	−3.50, 2.94
Full term (39^0/7^ to 40^6/7^)	382	0.13 (0.64)	−1.60, 3.25	0.19 (0.68)	−1.90, 2.49
Late term (41^0/7^ to 43^0/7^)	54	0.06 (0.65)	−1.07, 1.84	0.68 (0.74)	−0.56, 2.97
Weight‐for‐age *z*‐score					
Overall, term births (37^0/7^ to 43^0/7^)	727	0.43 (0.80)	−3.95, 3.13	0.39 (0.88)	−3.86, 3.03
Early term (37^0/7^ to 38^6/7^)	282	0.56 (0.80)	−2.13, 3.13	0.11 (0.85)	−2.45, 2.34
Full term (39^0/7^ to 40^6/7^)	389	0.35 (0.79)	−3.95, 2.67	0.50 (0.84)	−3.86, 3.03
Late term (41^0/7^ to 43^0/7^)	56	0.36 (0.81)	−1.31, 2.32	0.99 (0.86)	−0.84, 3.03
Head circumference‐for‐age *z*‐score					
Overall, term births (37^0/7^ to 43^0/7^)	719	0.01 (0.73)	−2.56, 2.39	−0.27 (0.83)	−2.53, 2.70
Early term (37^0/7^ to 38^6/7^)	279	0.09 (0.70)	−1.84, 1.92	−0.58 (0.72)	−2.53, 1.33
Full term (39^0/7^ to 40^6/7^)	385	−0.03 (0.74)	−2.06, 2.39	−0.14 (0.79)	−2.20, 2.51
Late term (41^0/7^ to 43^607^)	55	−0.06 (0.85)	−2.56, 2.06	0.36 (0.97)	−2.37, 2.70
ENID cohort					
Length‐for‐age *z*‐score					
Overall, term births (37^0/7^ to 43^0/7^)	441	−0.26 (1.06)	−4.44, 4.75	−0.25 (1.18)	−5.12, 5.38
Early term (37^0/7^ to 38^6/7^)	72	−0.15 (0.88)	−1.91, 2.72	−0.76 (0.89)	−2.55, 1.99
Full term (39^0/7^ to 40^6/7^)	244	−0.27 (1.04)	−3.06, 4.09	−0.30 (1.10)	−3.29, 3.90
Late term (41^0/7^ to 43^0/7^)	125	−0.30 (1.18)	−4.44, 4.75	0.16 (1.34)	−5.12, 5.38
Weight‐for‐age *z*‐score					
Overall, term births (37^0/7^ to 43^0/7^)	441	0.15 (1.02)	−4.65, 2.83	0.33 (1.07)	−4.56, 3.58
Early term (37^0/7^ to 38^6/7^)	72	0.40 (1.10)	−3.48, 2.70	−0.02 (1.12)	−3.81, 2.47
Full term (39^0/7^ to 40^6/7^)	244	0.17 (1.00)	−4.65, 2.82	0.32 (1.04)	−4.56, 2.91
Late term (41^0/7^ to 43^0/7^)	125	−0.03 (1.00)	−2.63, 2.83	0.54 (1.06)	−2.23, 3.58
Head circumference‐for‐age *z*‐score					
Overall, term births (37^0/7^ to 43^0/7^)	441	0.47 (1.13)	−3.89, 3.95	0.42 (1.21)	−4.61, 4.12
Early term (37^0/7^ to 38^6/7^)	72	0.70 (1.22)	−3.89, 2.97	0.04 (1.23)	−4.61, 2.35
Full term (39^0/7^ to 40^6/7^)	244	0.46 (1.13)	−3.01, 3.95	0.35 (1.18)	−2.95, 4.12
Late term (41^0/7^ to 43^0/7^)	125	0.36 (1.07)	−1.94, 3.01	0.78 (1.17)	−1.90, 3.62

**FIGURE 2:**
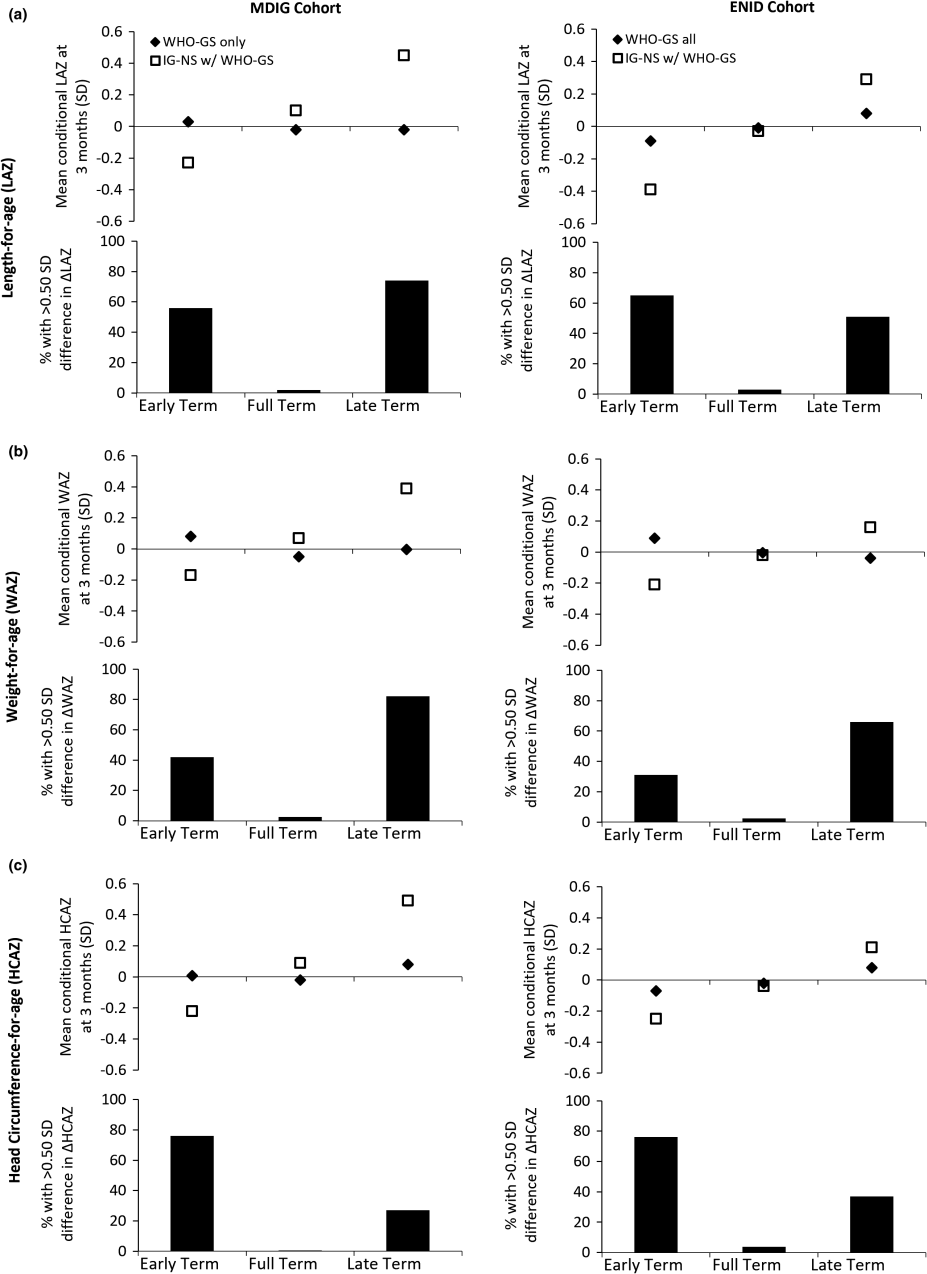
Mean conditional measures of growth between birth to 3 months of age and proportion of infants with between‐method difference in the change in *z*‐scores (Δ) greater than 0.5 SD for length‐for‐age (A), weight‐for‐age (B) and head circumference‐for‐age (C) among term‐born children using the WHO‐GS compared with the INTERGROWTH‐21st Newborn Size Standards (IG‐NS) at birth in conjunction with WHO‐GS at 3 months of age

Mean conditional growth measures at 3 months differed significantly between WHO‐GS and IG‐NS in all GA strata in both cohorts (Figure 2; Table S2). In both cohorts, distributions of conditional growth measures were similar across the GA strata using WHO‐GS but differed substantially across strata when using IG‐NS at birth (Figure 2; Table S2). In MDIG, early‐term infants appeared to grow slightly faster than the rest of the cohort in length, weight and head circumference using WHO‐GS, whereas the same group appeared to have substantially slower growth using IG‐NS at birth (Figure 2). The pattern was generally reversed in late‐term infants (Figure 2). Between‐method differences followed the same general pattern in ENID, such that use of IG‐NS at birth suggested relatively slower postnatal growth of early‐term infants compared with higher GA strata, but this was much less apparent (LAZ, HCAZ) or reversed (WAZ) when using WHO‐GS at birth (Figure 2). Differences in conditional growth between WHO‐GS and IG‐NS ranged widely from approximately −0.50 SD to 0.45 SD across anthropometric indices (Figure S4).

Differences in *z*‐scores between WHO‐GS and IG‐NS at birth were >0.2 SD for >60% of all infants across all anthropometric indices in both cohorts (Table S3). Overall, for 45%–50% of infants, the differences in *z*‐scores or growth between birth to 3 months when using WHO‐GS versus IG‐NS at birth were >0.32 SD, and for approximately 25% to 30% of the infants, differences were >0.50 SD (Table S3). Individual‐level differences in size and growth measures between WHO‐GS and IG‐NS were particularly marked in the early‐ and late‐term strata (Figures 2, S5 and S6).

## 4 COMMENT

### 4.1 Principal findings

In two longitudinal birth cohorts from Bangladesh and The Gambia, mean LAZ, WAZ and HCAZ, and the prevalence of undernutrition at birth among term‐born children were most similar when using WHO‐GS compared with IG‐NS among infants born between 39 and 40 weeks of GA. Mean anthropometric indices were systematically lower among early‐term infants and higher among late‐term infants when using WHO‐GS compared with IG‐NS at birth. The relatively slow growth of early‐term infants and relatively faster growth of late‐term infants observed when using IG‐NS at birth was not evident when using WHO‐GS as the only growth standard in longitudinal analyses. Importantly, the magnitude of changes in *z*‐scores in the first 3 months often differed at the individual‐level by more than pre‐specified thresholds (0.20 SD, 0.32 SD and 0.50 SD), highlighting the major effect of the choice of birth size standards on the assessment of an individual infant's growth trajectory, beyond its effects on population‐level inferences.

### 4.2 Strengths of the study

The strengths of the study are the use of data from two birth cohorts—one from South Asia and the other from sub‐Saharan Africa—to assess the consistency of inferences across populations with differing nutritional status. While this study could have been performed with simulated data, the use of two cohort datasets provides a real‐world illustration of the discrepancies between the two standards. In addition, ultrasound‐based assessments were used in both MDIG and ENID studies, which is the gold‐standard method for determining GA. Newborn and infant anthropometric measures were collected using robust methods in both studies.^6^

### 4.3 Limitations of the data

Although ultrasound assessments were used to confirm GA, these were not solely based on first trimester ultrasounds assessments, which are most reliable for GA dating. We restricted our analysis to only include data from liveborn infants at term gestation with no congenital anomalies, available GA and at least one available anthropometric measure collected within 48 h of birth to allow for robust methodological comparisons with high‐quality anthropometric data. Therefore, the sample population in this study may not be reflective of the general population in Bangladesh and The Gambia, yet inferences regarding the comparisons of anthropometric indices and indicators between WHO‐GS versus IG‐NS remain valid. Furthermore, the distribution of infants born across GA strata of early‐term, full‐term and late‐term was not equal which may have affected the ability to detect differences between anthropometric indices and indicators when using WHO‐GS versus IG‐NS within GA strata.

### 4.4 Interpretation

The INTERGROWTH‐21st newborn size standards (IG‐NS) were intended to replace previous size‐at‐birth references and to complement the WHO‐GS to enable the evaluation of the growth of a child or a group of children across a ‘continuum from the womb to the classroom’.^5^, ^15^, ^16^ Evaluating the implications of using IG‐NS relative to previous references or country‐specific national charts has been an active area of research^17^, ^18^, ^19^, ^20^, ^21^ and has generated thoughtful debate regarding the use of a single standard as opposed to population‐specific references, highlighting the importance of understanding the predictive utility of such standards/references in relation to health outcomes.^22^, ^23^, ^24^

The present study focused on examining the implications of variations in GA on newborn size and early growth among term infants only. Previous studies have consistently shown that perinatal and newborn mortality, morbidity and child development vary by week of GA even within the relatively narrow term duration of 37 to 42 weeks of GA.^25^, ^26^, ^27^, ^28^ The findings of this study add to this evidence base. Studies in which a large proportion of infants are born at early‐term or late‐term GAs will systematically have lower or higher estimated growth, respectively, when using IG‐NS at birth in conjunction with WHO‐GS postnatally, compared with the conventional use of the WHO‐GS as the sole standard applied to populations of term‐born babies at all ages. The degree to which measures of size and growth differ also depends on the nutritional status of the population, whereby discrepancies are expected to be larger in populations with a higher prevalence of foetal growth restriction, as was observed in the cohort from Bangladesh relative to The Gambia. In addition, the arithmetic difference in age‐ and sex‐adjusted anthropometric indices between two time points is one of the most commonly used metrics of growth in the literature.^29^ As such, epidemiologic research examining the relationship between growth in infancy and prenatal exposures or postnatal outcomes may be susceptible to bias due to artefactual, rather than biological, within‐child changes in *z*‐scores between birth and a postnatal time point when using IG‐NS at birth and WHO‐GS postnatally. This is particularly relevant in the first few months of life when variation in GA at birth is most likely to affect size and growth.^10^ Researchers may consider conducting sensitivity analyses using the WHO‐GS at birth to ensure that inferences using the IG‐NS are not susceptible to artefactual differences due to the use of different standards. Clinicians performing growth monitoring should similarly be aware that early postnatal growth trajectories of early‐ and late‐term infants may be substantially affected by the choice of neonatal size standard; however, further work is required to clarify the implications of the present findings in the clinical context and to assess the validity of early infant growth trajectories when using WHO‐GS versus IG‐NS at birth in conjunction with WHO‐GS postnatally to detect associations with risk factors or postnatal outcomes.

## 5 CONCLUSIONS

The INTERGROWTH‐21st newborn size standards and the WHO‐GS are not interchangeable at birth for all the GAs in the range in which they overlap (i.e. 37to 42 weeks), especially for children born at <39 weeks or >40 weeks of GA. Using IG‐NS at birth in conjunction with WHO‐GS at a postnatal time point in the same analysis may yield within‐child changes in anthropometric *z*‐scores that are an artefact of combining two different standards, particularly among early‐ and late‐term infants. Therefore, researchers should carefully evaluate the interchangeability of *z*‐score values when using IG‐NS compared with the WHO‐GS at birth among term‐born children, and explicitly assess the potential implications for summarising early life growth trajectories and robustness of inferences for epidemiologic associations.

## CONFLICT OF INTEREST

None to declare.

## Author Contribution

NP and DER conceptualized the study; NP, EOO, AMP, PSS, SEM, and DER contributed to the study design; AM, AMP, SEM, and DER contributed to acquisition of the data used in the study; EOO contributed to the analysis; NP led the analysis and interpretation of the data and drafted the manuscript. All authors revised the manuscriptcritically for important intellectual content and provided their final approval of the version to be published.

## Supporting information

Supinfo S1

## Data Availability

The data that support the findings of this study are available upon reasonable request from Dr. Daniel Roth (The Maternal Vitamin D for Infant Growth trial) and from Dr. Sophie Moore (The Early Nutrition and Immune Development trial). The data are not publicly available due to privacy or ethical restrictions.
