# Supplementary material for: Implications for quantifying early life growth trajectories of term‐born infants using INTERGROWTH‐21st newborn size standards at birth in conjunction with World Health Organization child growth standards in the postnatal period
Source: Paediatr Perinat Epidemiol. 2022 May 16;36(6):839–50. doi: 10.1111/ppe.12880 (PMC9790258; doi:10.1111/ppe.12880)
Supplement: Supplementary file 1 — Supinfo S1 [file PPE-36-839-s001.pdf]

Implications for quantifying early life growth trajectories of term-born infants using INTERGROWTH-21st newborn size standards at birth in conjunction with World Health Organization child growth standards in the postnatal period

Supplementary Material Online

Nandita Perumal, Eric O. Ohuma, Andrew M. Prentice, Prakesh S. Shah, Abdullah Al Mahmud, Sophie E. Moore, Daniel E. Roth

Table S1: Baseline maternal characteristics of term-born infants included in this analysis from MDIG and ENID studies.

**Maternal characteristics**	**MDIG Analytical sample (n = 755)**	**ENID Analytical sample (n = 522)**
Age, years		
Median	22	29.8
Range	18-39	17.2-47.2
Education level[1], n (%)		
No school	30 (3.97)	396 (77)
Primary school or incomplete secondary school	551 (73)	90 (17.5)
Secondary school complete or higher	174 (23)	28 (5.5)
Married, n (%)	750 (99)	522 (100)
Gravidity, no. of pregnancies (including current)		
Median	2	4
Range	1-9	1-10
Maternal height (cm), mean ± SD	151 ± 5.3	161.9 ± 5.8

[1] Maternal education was missing for 8 participants in the ENID study; therefore, the proportions are out of total of 514 mothers.

Table S2: Length-for-age, weight-for-age, and head circumference for age z-scores at 3 months among term-born children using the World Health Organization Child Growth Standards.

Anthropometric indices	3 months		
	n	Mean (SD)	Range min, max
**MDIG Cohort**			
**Length-for-age z score**			
Overall, term births ($37^{0/7} – 43^{0/7}$)	709	-0.85 (0.89)	-3.48, 1.97
Early term ($37^{0/7}$ to $38^{6/7}$)	273	-1.04 (0.89)	-3.45, 1.39
Full term ($39^{0/7}$ to $40^{6/7}$)	382	-0.75 (0.87)	-3.48, 1.97
Late term ($41^{0/7}$ to $43^{0/7}$)	54	-0.61 (0.83)	-2.50, 1.27
**Weight-for-age z score**			
Overall, term births ($37^{0/7} – 43^{0/7}$)	727	-0.83 (0.93)	-4.37, 1.75
Early term ($37^{0/7}$ to $38^{6/7}$)	282	-0.87 (0.94)	-3.77, 1.73
Full term ($39^{0/7}$ to $40^{6/7}$)	389	-0.82 (0.91)	-4.37, 1.75
Late term ($41^{0/7}$ to $43^{0/7}$)	56	-0.70 (0.99)	-3.66, 1.61
**Head circumference-for-age z score**			
Overall, term births ($37^{0/7} – 43^{0/7}$)	719	-0.89 (0.85)	-3.95, 2.66
Early term ($37^{0/7}$ to $38^{6/7}$)	279	-1.01 (0.82)	-3.48, 1.60
Full term ($39^{0/7}$ to $40^{6/7}$)	385	-0.86 (0.85)	-3.95, 1.56
Late term ($41^{0/7}$ to $43^{0/7}$)	55	-0.56 (0.93)	-2.35, 2.66
**ENID Cohort**			
**Length-for-age z score**			
Overall, term births ($37^{0/7} – 43^{0/7}$)	443	-0.35 (1.07)	-3.29, 4.45
Early term ($37^{0/7}$ to $38^{6/7}$)	72	-0.66 (0.98)	-3.12, 1.54
Full term ($39^{0/7}$ to $40^{6/7}$)	245	-0.36 (1.04)	-3.29, 4.45
Late term ($41^{0/7}$ to $43^{0/7}$)	126	-0.14 (1.15)	-3.02, 4.40
**Weight-for-age z score**			
Overall, term births ($37^{0/7} – 43^{0/7}$)	443	-0.53 (1.04)	-4.57, 2.13
Early term ($37^{0/7}$ to $38^{6/7}$)	72	-0.62 (1.18)	-4.57, 1.75
Full term ($39^{0/7}$ to $40^{6/7}$)	245	-0.56 (0.99)	-3.79, 2.13
Late term ($41^{0/7}$ to $43^{0/7}$)	126	-0.42 (1.06)	-3.51, 2.05
**Head circumference-for-age z score**			
Overall, term births ($37^{0/7} – 43^{0/7}$)	443	-0.40 (0.91)	-3.19, 2.95
Early term ($37^{0/7}$ to $38^{6/7}$)	72	-0.61 (0.96)	-3.19, 1.74
Full term ($39^{0/7}$ to $40^{6/7}$)	245	-0.43 (0.89)	-2.71, 2.19
Late term ($41^{0/7}$ to $43^{0/7}$)	126	-0.24 (0.90)	-2.18, 2.95

Table S3: Mean conditional measures of growth among term-born children using the WHO-GS vs. INTERGROWTH 21st Neonatal Standards at birth in conjunction with WHO-GS at 3 months of age[a].

Anthropometric indices	n	Mean conditional measures of growth at 3 months of age: WHO-GS at birth and WHO-GS at 3 months of age		IG-NS at birth and WHO-GS at 3 months of age	
		Mean (SD)	Range min, max	Mean (SD)	Range min, max
**MDIG Cohort**					
**Length-for-age z score**					
Early term ($37^{0/7}$ to $38^{6/7}$)	273	0.03 (0.63)	-2.58, 2.64	-0.23 (0.64)	-2.93, 1.61
Full term ($39^{0/7}$ to $40^{6/7}$)	382	-0.02 (0.57)	-1.65, 1.96	0.10 (0.58)	-1.52, 1.90
Late term ($41^{0/7}$ to $43^{0/7}$)	54	-0.02 (0.60)	-1.31, 1.79	0.45 (0.64)	-0.89, 2.59
**Weight-for-age z score**					
Early term ($37^{0/7}$ to $38^{6/7}$)	282	0.08 (0.76)	-2.68, 1.95	-0.17 (0.78)	-2.88, 1.67
Full term ($39^{0/7}$ to $40^{6/7}$)	389	-0.05 (0.75)	-4.10, 2.26	0.07 (0.77)	-3.94, 2.57
Late term ($41^{0/7}$ to $43^{0/7}$)	56	-0.003 (0.80)	-2.10, 2.07	0.39 (0.83)	-1.93, 2.50
**Head circumference-for-age z score**					
Early term ($37^{0/7}$ to $38^{6/7}$)	279	0.007 (0.61)	-1.53, 1.71	-0.22 (0.62)	-1.94, 1.49
Full term ($39^{0/7}$ to $40^{6/7}$)	385	-0.02 (0.66)	-1.98, 2.28	0.09 (0.68)	-1.88, 2.47
Late term ($41^{0/7}$ to $43^{0/7}$)	55	0.08 (0.79)	-2.14, 2.37	0.49 (0.82)	-1.79, 2.93
**ENID Cohort**					
**Length-for-age z score**					
Early term ($37^{0/7}$ to $38^{6/7}$)	72	-0.09 (0.78)	-1.66, 1.81	-0.39 (0.79)	-1.99, 1.41
Full term ($39^{0/7}$ to $40^{6/7}$)	244	-0.01 (0.92)	-2.62, 4.56	-0.03 (0.93)	-2.57, 4.53
Late term ($41^{0/7}$ to $43^{0/7}$)	125	0.08 (1.04)	-2.57, 4.88	0.29 (1.07)	-2.43, 5.11
**Weight-for-age z score**					
Early term ($37^{0/7}$ to $38^{6/7}$)	72	0.09 (1.05)	-3.83, 2.42	-0.21 (1.05)	-4.08, 2.22
Full term ($39^{0/7}$ to $40^{6/7}$)	244	-0.005 (0.91)	-4.07, 2.67	-0.02 (0.91)	-4.02, 2.62
Late term ($41^{0/7}$ to $42^{6/7}$)	125	-0.04 (0.93)	-2.77, 2.64	0.16 (0.94)	-2.60, 2.89
**Head circumference-for-age z score**					
Early term ($37^{0/7}$ to $38^{6/7}$)	72	-0.07 (0.94)	-3.27, 1.98	-0.25 (0.93)	-3.36, 1.85
Full term ($39^{0/7}$ to $40^{6/7}$)	244	-0.02 (0.83)	-2.28, 2.56	-0.04 (0.84)	-2.16, 2.50
Late term ($41^{0/7}$ to $42^{6/7}$)	125	0.08 (0.82)	-1.88, 2.61	0.21 (0.82)	-1.75, 2.75

[a] Overall mean conditional measures for all term-born infants (i.e. $37^{0/7}$ to $43^{0/7}$) are expected to be zero by definition.

Table S4: Individual-level differences in anthropometric indices among term-born children using the World Health Organization Child Growth Standards (WHO-GS) at birth and 3 months of age versus INTERGROWTH 21st Newborn Size Standards (IG-NS) at birth with WHO-GS at 3 months of age.

Anthropometric indices	Infants for whom the difference between WHO-GS and IG-NS z-scores at birth was greater than the specified threshold, n (%)				Infants with Δ z-scores between 0 to 3 months that differed by greater than the specified threshold when WHO-GS vs IG-NS is used at birth (with WHO-GS at 3 months), n (%)			
	n	>0.20 SD	>0.32 SD	>0.5 SD	n	>0.20 SD	>0.32 SD	>0.5 SD
**MDIG Cohort**								
**Length-for-age z score <-2 SD, n (%)**								
Overall ($37^{0/7}$ – $42^{6/7}$)	744	483 (65)	351 (47)	210 (28)	709	456 (64)	328 (46)	196 (28)
Early term ($37^{0/7}$ to $38^{6/7}$)	286	274 (96)	220 (77)	157 (55)	273	261 (96)	209 (77)	149 (55)
Full term ($39^{0/7}$ to $40^{6/7}$)	396	147 (37)	72 (18)	7 (1.8)	382	141 (37)	68 (18)	7 (1.8)
Late term ($41^{0/7}$ to $42^{6/7}$)	62	62 (100)	59 (95)	46 (74)	54	54 (100)	51 (94)	40 (74)
**Weight-for-age z score <-2 SD, n (%)**								
Overall ($37^{0/7}$ – $42^{6/7}$)	753	475 (63)	335 (45)	182 (24)	727	457 (63)	319 (44)	175 (24)
Early term ($37^{0/7}$ to $38^{6/7}$)	292	240 (82)	184 (63)	121 (41)	282	232 (82)	177 (63)	119 (42)
Full term ($39^{0/7}$ to $40^{6/7}$)	398	172 (43)	90 (23)	10 (2.5)	389	169 (43)	88 (23)	10 (2.6)
Late term ($41^{0/7}$ to $42^{6/7}$)	63	63 (100)	61 (97)	51 (81)	56	56 (100)	54 (96)	46 (82)
**Head circumference-for-age z score <-2 SD, n (%)**								
Overall ($37^{0/7}$ – $42^{6/7}$)	748	500 (67)	397 (53)	242 (32)	719	477 (66)	379 (53)	230 (32)
Early term ($37^{0/7}$ to $38^{6/7}$)	290	290 (100)	290 (100)	223 (77)	279	279 (100)	279 (100)	213 (76)
Full term ($39^{0/7}$ to $40^{6/7}$)	395	155 (39)	65 (17)	2 (0.51)	385	151 (39)	64 (17)	2 (0.52)
Late term ($41^{0/7}$ to $42^{6/7}$)	63	55 (87)	42 (67)	17 (27)	55	47 (86)	36 (66)	15 (27)
**ENID Cohort**								
**Length-for-age z score <-2 SD, n (%)**								
Overall ($37^{0/7}$ – $42^{6/7}$)	518	336 (65)	245 (47)	132 (26)	441	293 (66)	215 (49)	118 (27)
Early term ($37^{0/7}$ to $38^{6/7}$)	79	78 (99)	73 (92)	51 (65)	72	72 (100)	67 (93)	47 (65)
Full term ($39^{0/7}$ to $40^{6/7}$)	289	128 (44)	55 (19)	7 (2.4)	244	110 (45)	47 (19)	7 (2.9)
Late term ($41^{0/7}$ to $42^{6/7}$)	150	130 (87)	117 (78)	74 (49)	125	111 (89)	101 (81)	64 (51)
**Weight-for-age z score <-2 SD, n (%)**								
Overall ($37^{0/7}$ – $42^{6/7}$)	519	347 (67)	248 (48)	127 (25)	442	302 (68)	216 (49)	111 (25)
Early term ($37^{0/7}$ to $38^{6/7}$)	78	68 (87)	51 (65)	23 (30)	71	63 (89)	46 (65)	22 (31)
Full term ($39^{0/7}$ to $40^{6/7}$)	290	132 (46)	63 (22)	7 (2.4)	245	115 (47)	55 (23)	6 (2.5)
Late term ($41^{0/7}$ to $42^{6/7}$)	151	147 (97)	134 (89)	97 (64)	126	124 (98)	115 (91)	83 (66)
**Head circumference-for-age z score <-2 SD, n (%)**								
Overall ($37^{0/7}$ – $42^{6/7}$)	519	327 (63)	220 (42.4)	122 (24)	443	282 (64)	193 (44)	111 (25)
Early term ($37^{0/7}$ to $38^{6/7}$)	79	79 (100)	79 (100.0)	60 (76)	72	72 (100)	72 (100)	55 (76)
Full term ($39^{0/7}$ to $40^{6/7}$)	289	123 (43)	45 (15.6)	10 (3.5)	245	102 (42)	38 (16)	9 (3.7)
Late term ($41^{0/7}$ to $42^{6/7}$)	151	125 (83)	96 (63..6)	52 (34)	126	108 (86)	83 (66)	47 (37)

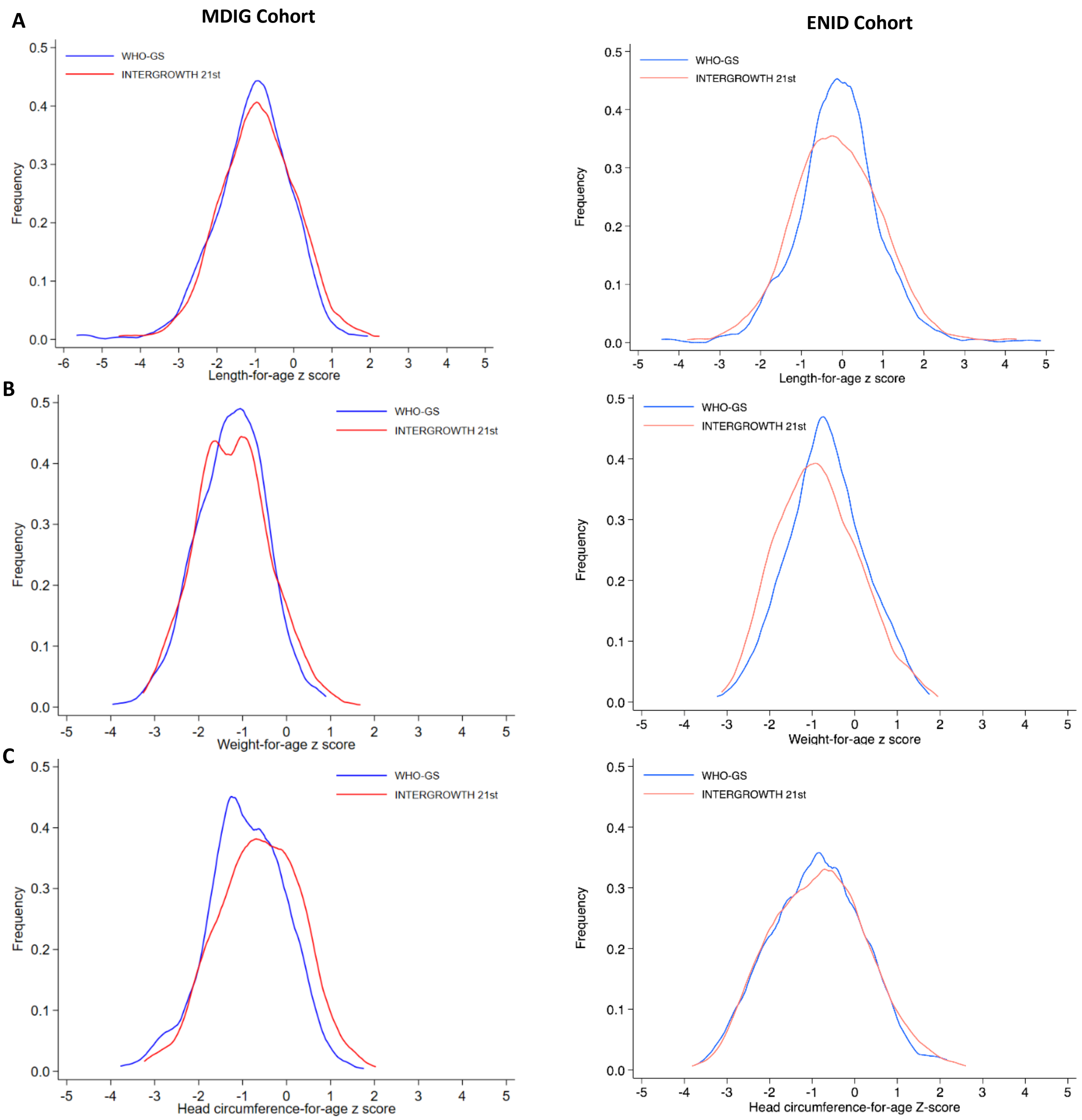

Figure S1: Distribution of birth length-for-age, weight-for-age, and head circumference for age z-scores among term-born children using the World Health Organization Child Growth Standards (WHO-GS) compared to the INTERGROWTH 21st Newborn Size Standards (IG-NS) at birth.

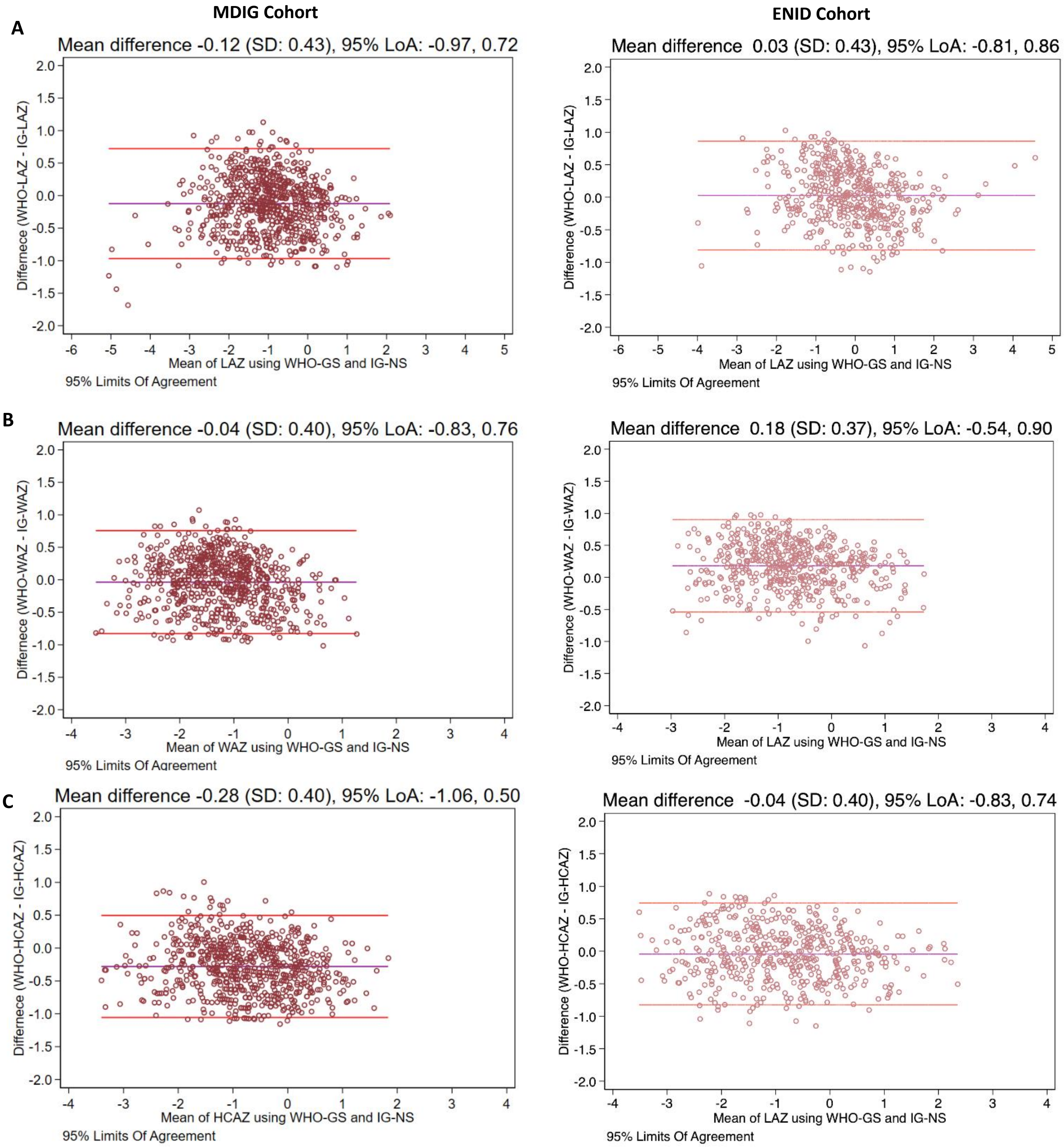

Figure S2: Bland-Altman plots of birth length-for-age (LAZ), weight-for-age (WAZ), and head circumference for age z-scores (HCAZ) among term-born children using the World Health Organization Child Growth Standards (WHO-GS) compared to the INTERGROWTH 21st Newborn Size Standards (IG-NS) at birth.

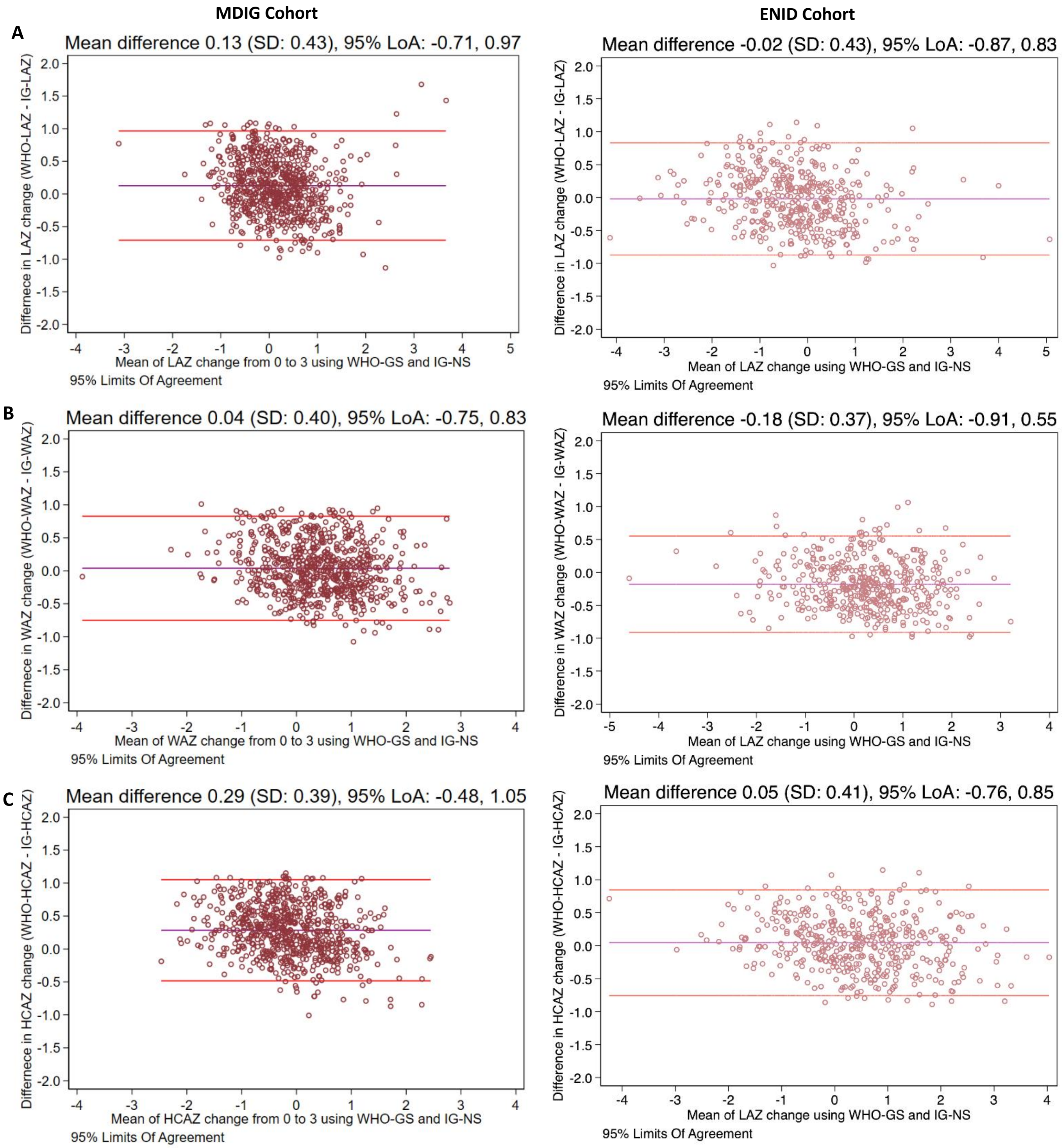

Figure S3: Bland-Altman plots of mean change in length-for-age (LAZ), weight-for-age (WAZ), and head circumference-for-age z-score (HCAZ) from birth to 3 months of age among term-born children using the World Health Organization Child Growth Standards (WHO-GS) compared to the INTERGROWTH 21st Newborn Size Standards (IG-NS) at birth in conjunction with the WHO-GS postnatally.

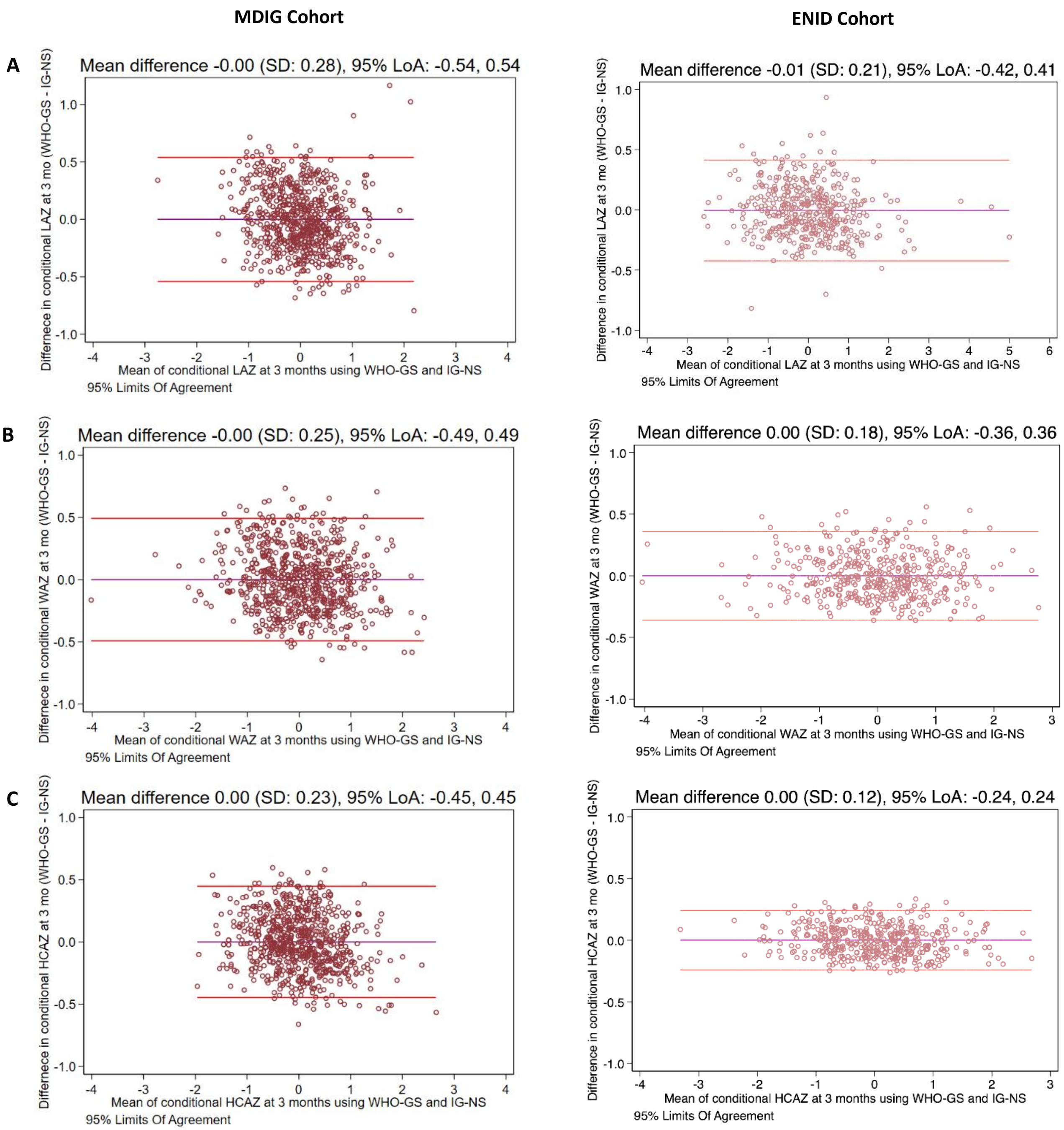

Figure S4: Bland-Altman plots of conditional length-for-age (LAZ), weight-for-age (WAZ), and head circumference-for-age z-score (HCAZ) from at 3 months of age among term-born children using the World Health Organization Child Growth Standards (WHO-GS) compared to the INTERGROWTH 21st Newborn Size Standards at birth in conjunction with the WHO-GS postnatally.

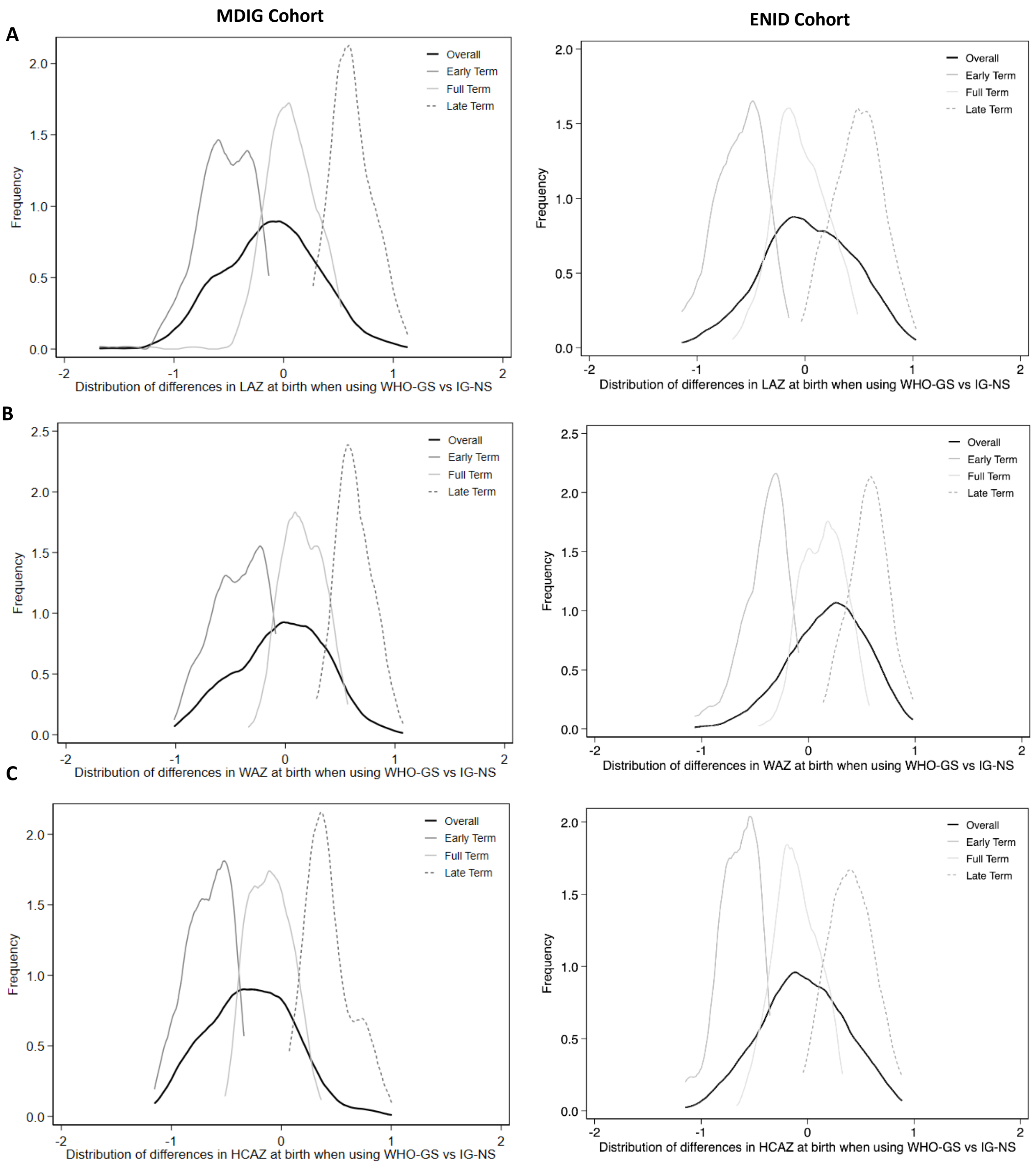

Figure S5: Distribution of differences in length-for-age (A), weight-for-age (B), and head circumference-for-age z-scores (C) at birth using the World Health Organization Child Growth Standards (WHO-GS) compared to the INTERGROWTH 21st Newborn Size Standards compared, overall and by gestational age strata.

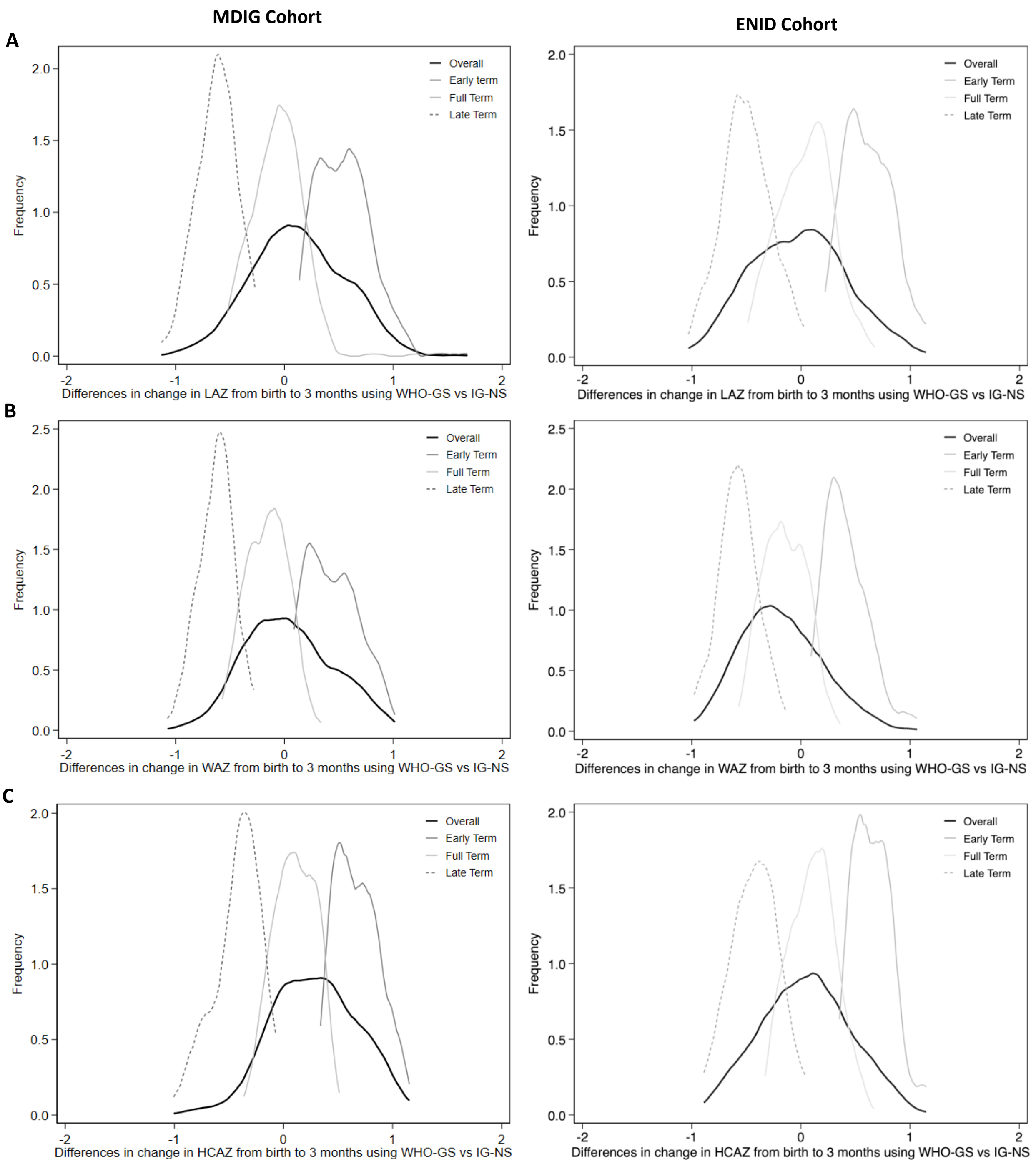

Figure S6: Differences in change in length-for-age z-score (A), weight-for-age (B), and head circumference-for-age (C) z-scores from birth to 3 months of age among term-born children when using the World Health Organization Child Growth Standards (WHO-GS) compared to the INTERGROWTH 21st Newborn Size Standards (IG-NS) at birth and the WHO-GS at 3 months postnatal age, overall and by gestational age strata.
